# Letter in response to “Cherry-picking of evidence fails to accurately show extent of overdiagnosis: Mammographic screening harms understated”

**DOI:** 10.1002/jmrs.11

**Published:** 2013-06-04

**Authors:** Warwick Lee, Gudrun Peters

**Affiliations:** BreastScreen NSW, Cancer Institute NSWPO Box 41, Alexandria, NSW, 1435, Australia; BreastScreen TasmaniaLevel 4, 25 Argyle Street, Hobart, TAS, 7001, Australia

Re: Woolley E. Cherry-picking of evidence fails to accurately show extent of overdiagnosis: Mammographic screening harms understated. *J Med Radiat Sci* 2013; **60**(2): 71.

We thank Ms. Woolley for her comments which provide the opportunity to refer to the excellent Independent Breast Screening Review (IBSR)^1^,^2^ by the Independent U.K. Panel on Breast Cancer Screening, led by Prof. Sir Michael Marmot. This review was not published at the time of submission of our review. The IBSR Panel is truly expert and independent as detailed in the full report.^2^ None of the panel members have previously published on breast cancer screening. Importantly, a patient advocate (Maggie Wilcox^3^) was a member of the panel. The key findings of the IBSR were that review of the evidence regarding mammographic screening “suggested a 20% reduction in mortality in women invited to participate in a 20-year screening programme” and that “1 breast cancer death was averted for every 235 women invited to screening and one death averted for every 180 women who attend screening.” This compares to the estimate of Gotzsche and Nielson^4^ of one breast cancer death averted in 2000 women invited to screening for 10 years and the estimate of Duffy et al.^5^ of one death averted for every 113 women screened for a 20 year period. The estimate of overdiagnosis by the panel was 19% of cancers diagnosed in women screened and 11% on a population basis. This estimate is similar to the estimate of the EUROSCREEN Working Group^6^ of between 1% and 10% on a population basis. From the perspective of an individual woman, the IBSR^2^ stated that a woman screened “has perhaps a 1% chance of having a cancer diagnosed, and treated with surgery and other modalities, that would never have caused problems had she not been screened.” The IBSR estimated that one cancer death is prevented for three cancer cases overdiagnosed and treated.^1^,^2^ This compares to the estimate by Duffy et al.^5^ of 2–2.5 lives saved for each case of overdiagnosis, referred to in our review, and the estimate of Gotzsche and Nielson,^4^ not referred to in our review, of one life saved for 10 women overdiagnosed and treated. Based on the IBSR, the figure of Duffy et al.^5^ may be an underestimate, as indicated by Ms. Woolley, but the figure of Gotzsche and Nielson^4^ should be considered an overestimate. An editorial in *Lancet*, commenting on the IBSR, states that the review should begin to lay the benefits versus harm controversy to rest.^7^ The editorial further states, “The Panel's report, the latest and best available systematic review, shows that the U.K. breast-screening programme extends lives and that, overall, the benefits outweigh the harms.”
